# Stricturing Phenotype Predisposes to Small Bowel and Colorectal Malignancy in Crohn's Disease

**DOI:** 10.1002/jgh3.70401

**Published:** 2026-05-18

**Authors:** Kiran Josy Kanjamala, Prakash Zacharias, Shibi Mathew, Hasim Ahamed, Remya R. Pai, Anvin Kurian Thomas, Arjun R. Gupta, Mathew Philip

**Affiliations:** ^1^ Lisie Institute of Gastroenterology, Hepatology and Transplantation (LIGHT), Lisie Hospital Kochi Kerala India

**Keywords:** anal canal neoplasm, colorectal carcinoma, inflammatory bowel disease, intestinal strictures, small bowel carcinoma

## Abstract

**Background:**

Carcinogenesis is a major complication in patients with inflammatory bowel disease (IBD). We report the data of Crohn's disease (CD)‐associated intestinal malignancy in our IBD cohort.

**Methods:**

Retrospective analysis of data from prospectively maintained medical records of 1416 CD patients diagnosed or under follow up from January 1st 2013 to December 31st 2023.

**Results:**

A total of 1416 patients were included (13037.9 person‐years of follow‐up with mean age at diagnosis 27.62 ± 11.8 years, 64.1% males, disease duration: 9.2 ± 6.7 years). Eighteen patients (1.27%) developed intestinal malignancy at a mean age of 46.7 ± 14.2 years after 15.9 ± 10.3 years of disease. Colorectal carcinoma (10/1416) was most frequent, followed by jejunal (4/1416), anal canal (3/1416), and ileal (1/1416) cancers. All luminal malignancies occurred in stricturing disease, while anal canal carcinoma occurred in chronic perianal fistulizing disease with anal stricture. The cumulative risk of malignancy in stricturing disease was 1.5%, 8.3%, and 25.1% in the first, second, and third decades of disease, respectively. Independent risk factors included older age at CD diagnosis, longer disease duration, stricturing phenotype, and active smoking. The standardized incidence ratio (SIR) for intestinal malignancy was 95.36 (95% CI: 52.98–143.04), nearly 95‐fold higher than the general population.

**Conclusion:**

The incidence rate of intestinal malignancy was 1.27% in our CD cohort with standardized incidence rate of 95.3. Stricturing disease predisposed to small bowel and colorectal carcinoma. Carcinoma in anal canal was seen in chronic perianal fistulizing disease with anal canal stricture.

## 1 Introduction

The incidence of inflammatory bowel disease (IBD) in India saw a significant increase between 1990 and 2019, the total number of patients doubling [1] with Indians having the highest incidence and prevalence of IBD among all Asian countries [2]. As disease prevalence rises and chronic intestinal inflammation persists over time, gastroenterologists are increasingly encountering serious complications, including malignancies, in both ulcerative colitis (UC) and Crohn's disease (CD). It has been shown in various studies that patients with both UC and CD have an increased risk of bowel cancer and is usually associated with poor prognosis [3]. The occurrence of colorectal cancer (CRC) in UC was first described by Crohn's and colleagues in 1925 [4] and it was Warren and Sommers 23 years later, in 1948, who reported the first case of CRC in CD then termed as regional enteritis [5]. Ginzburg reported the first case of small bowel adenocarcinoma (SBA) in CD in 1956 [6]. Since CD can affect any portion of the gastrointestinal tract from mouth to anus, all these areas are prone to malignant changes.

In a recent meta‐analysis by Uchino et al., the standardized incidence ratios (95% confidence intervals [CIs]) of CRC were 2.08 (1.43–3.02) and small bowel cancer (SBC) were 22.01 (9.10–53.25) in CD. The prevalence of CRC and SBC was 0.77% and 0.23%, respectively, during a median follow‐up of 12.55 years. The incidence of carcinoma in the anal canal was about 0.7% in patients with perianal Crohn's disease [7]. Although there are studies reporting the risk of colorectal carcinoma in UC, only few studies report the risk of intestinal malignancies in CD in India as well as Asia as compared to the West. We aimed to delineate the prevalence and risk factors for intestinal malignancy in CD by a comprehensive evaluation of the data from our cohort.

## 2 Methods

This study was a retrospective longitudinal cohort analysis of a prospectively maintained database of patients with IBD. We analyzed medical records of patients with CD diagnosed according to established standard criteria [8] at Lisie Institute of Gastroenterology, Hepatology and Transplantation (LIGHT), Lisie Hospital, Kochi, India.

### 2.1 Study Population

All patients with CD who were diagnosed or were under follow up from January 1st 2013 to December 31st 2023 at IBD clinic, LIGHT. Medical records of patients were followed up from the date of IBD diagnosis until either the date of first cancer diagnosis or December 31st 2023. Study participants lost to follow‐up were censored at the date of their last contact, with their disease duration calculated up to that point only. This study was approved by the Institutional Ethics Committee with institutional review board number LISIE/IRB/JULY‐2‐24/03.

### 2.2 Study Design and Characteristics

This was a retrospective cohort study of IBD database maintained prospectively which includes a comprehensive history, examination findings, relevant investigations, and serial symptom assessments of patients. In order to assess the occurrence and the cumulative risk of bowel malignancy in CD and the risk factors, the following data were collected—(a) patient demographic parameters, (b) age of diagnosis of CD and malignancy, (c) duration of disease, (d) phenotype and extent of CD, (e) medical therapy, (f) previous history of anti‐tuberculous therapy (ATT), (g) family history of bowel malignancy, (h) family history of IBD (first‐degree relatives), (i) history of primary sclerosing cholangitis (PSC), (j) smoking status—recorded as active smoking only when patients continued to smoke after Crohn's disease diagnosis, and (k) history of bowel surgery.

### 2.3 Objectives

In this study, the primary objective was assessment of the incidence rate of intestinal malignancy as well as the standardized incidence ratio (SIR) in Indian patients with CD. Secondary objectives were (i) identification of risk factors, (ii) the various sites of malignancy in the bowel, and (iii) the cumulative risk of developing intestinal malignancy in CD patients.

### 2.4 Statistical Analysis

Data were collected in Microsoft Excel and analyzed using IBM SPSS Version 25. Descriptive statistics were used to summarize the baseline characteristics of the study population. Categorical variables were presented as frequencies and percentages, while continuous variables were summarized using means and standard deviation. Chi‐Square tests were conducted to assess associations between categorical risk factors and the presence of malignancy. To estimate the cumulative risk of developing intestinal malignancy over time, time‐to‐event analysis was performed, and cumulative hazard functions were generated using the Nelson–Aalen estimator. To assess the impact of multiple covariates on the risk of developing malignancy, a Cox Proportional Hazards Model was applied. The model evaluated the hazard ratio (HR), which was reported with a 95% CI, and statistical significance was assessed with a *p* value of < 0.05.

The SIR, defined as the ratio of observed to expected cancer cases, was employed to estimate the relative risk of intestinal malignancy in individuals with CD. Confidence intervals (CIs) for the SIR were calculated using a standard statistical formula [9].

SIR computation involved the following steps:

- *Step 1*: Incidence rate = (Number of intestinal malignancies in Crohn's disease cohort/Total number of Crohn's disease patients) × 100
- *Step 2*: This rate was converted to per hundred thousand population by multiplying by 1000.
- *Step 3*: SIR = (Observed incidence in Crohn's disease cohort)/(Expected incidence in the general population)

For expected incidence, national data from 2022 were used, estimating approximately 194 093 incident intestinal malignancy cases in India, corresponding to a crude incidence rate of 13.33 per 100 000 population [10].

## 3 Results

### 3.1 Baseline Demographics of the Cohort (Table 1)

**TABLE 1:** Baseline demographics of the cohort (*N* = 1416).

Mean age of diagnosis ± SD (years)	27.62 ± 11.8 years
Mean duration of disease	9.2 ± 6.7 years
Males/females	908 (64.1%) *I* 508 (35.9%)
Disease location	Ileal (L1)—276 (19.5%)
	Colonic (L2)—225 (15.9%)
	Ileocolonic (L3)—754 (53.2%)
Isolated upper gastrointestinal/small bowel	(L4)‐161 (11.4%)
	L4a—20
	L4b—131
	L4a + L4b—10
Disease phenotype	
Non—stricturing, non‐penetrating (B1)	1031 (73.7%)
Stricturing (B2)	261 (17.7%)
Penetrating (B3)	71 (5%)
Stricturing and penetrating (B2B3)	53 (3.7%)
Perianal disease	
Fistula	282 (20.2%)
Fissure	117 (8.4%)
Stricture	22 (1.6%)
Skin tag	2 (0.1%)
Anal ulcer	9 (0.6%)
Abscess	51 (3.6%)
Medications ongoing%	
Azathioprine	1272 (90%)
Methotrexate	29 (2%)
Biologics	239 (16.9%)
Adalimumab	100 (41.8%)
Infliximab	127 (53.1%)
Vedolizumab	11 (4.6%)
Ustekinumab	5 (2%)
Family history of intestinal malignancy	18 (1.3%)
Family history of IBD	134 (9.6%)
Smoking history	27 (1.9%)
Previous ATT	179 (12.6%)
Primary sclerosing cholangitis	0
History of bowel surgery	380 (26.8%)

A total of 1416 CD patients who were under follow up during the study period were included for data analysis. The mean age of the study population at diagnosis of CD was 27.62 ± 11.8 years. Males constituted 64.1% of the study population. Total disease duration was 13037.9 person‐years with a mean duration of 9.2 ± 6.7 years. The disease location was ileal (L1)—276 (19.5%), colonic (L2)—225 (15.9%), and ileocolonic (L3)—754 (53.2%). Isolated upper gastrointestinal and small bowel (L4) involvement was seen in 161 (11.4%). Involvement up to ligament of Treitz (L4a) was seen in 20, extent up to middle third ileum (L4b) in 131 and both L4a + L4b in 10 patients respectively. Most common disease phenotype was non—stricturing, non‐penetrating (B1)—1033 (73%), followed by stricturing (B2)—259 (18.3%), penetrating (B3)—71 (5%), and stricturing and penetrating B2B3—53 (3.7%), respectively. Perianal involvement was seen in 483 patients (31.8%). Family history of intestinal malignancy was seen in 18 (1.3%) and family history of IBD in 134 patients (9.5%).

### 3.2 Characteristics of Patients With Intestinal Malignancy (Table 2)

**TABLE 2:** Characteristics of patients with intestinal malignancy.

Mean age of diagnosis+/‐SD (years)	35.7 ± 14.7
Mean duration of disease at diagnosis of malignancy (years)	15.9 ± 10.3
Males/females	10 (55.6%)/ 8 (44.4%)
Disease phenotype (excluding anal canal carcinoma)	
Non‐stricturing, non‐penetrating (B1)	0 (0%)
Stricturing (B2)	13 (86.7%)
Penetrating (B3)	0 (0%)
Stricturing and penetrating (B2B3)	2 (13.3%)
Anal canal carcinoma	
Perianal stricture	3/22 (13.6%)
Family history of intestinal malignancy	1 (5.6%)
Family history of IBD	0
Smoking history	2 (11.1%)
Previous ATT	5 (27.8%)
Primary sclerosing cholangitis	0
History of previous bowel surgery	0

**Abbreviation:** SD, Standard deviation.

**FIGURE 1:**
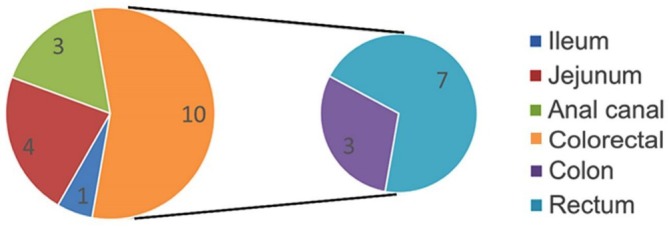
Location of intestinal malignancy.

Eighteen (1.27%) developed intestinal malignancy in a mean disease duration of 15.9 ± 10.3 years. The incidence density of intestinal malignancy was 1.379 per 1000 person‐years, corresponding to an annual incidence rate of approximately 0.13%. Mean age at diagnosis of malignancy was 46.7 ± 14.2 years. The sites of malignancy in descending order were colorectal carcinoma (10/1416), which was most frequent, followed by jejunal (4/1416), anal canal (3/1416), and ileal (1/1416) cancers (Figure 1). Of these patients, four had malignancy at index presentation, two each in jejunum and colon. Histopathology of all malignancies was adenocarcinoma except for a rectal lymphoma. All cases of luminal intestinal carcinoma occurred exclusively in patients with a stricturing disease phenotype, whereas anal canal carcinoma developed in those with chronic fistulizing disease associated with anal canal stricture.

The SIR of intestinal malignancy in our CD cohort was 95.36 (95% CI: 52.98–143.04) indicating that the observed risk of intestinal malignancy is approximately 95 times higher than expected based on general population rates. When site‐specific risks were examined, colonic carcinoma exhibited a markedly elevated incidence with an SIR of 161 (95% CI: 72–359), while rectal carcinoma had an SIR of 79.4 (95% CI: 25.6–246.3). The relative risk was strikingly elevated for anal canal carcinoma, which demonstrated an SIR of 595 (95% CI: 192–1836). Small‐intestinal carcinoma also showed a significant elevation, with an SIR of 97.1 (95% CI: 40.4–233.4), highlighting the considerable vulnerability of the small bowel to malignant transformation in this cohort. The clinical profile and outcome of patients with malignancy has been depicted in Table 3, respectively.

**TABLE 3:** Clinical profile of patients with intestinal malignancy.

Serial number	Gender	Age of diagnosis of disease	Age of diagnosis of malignancy	Montreal classification	Smoking	Family history of IBD	Family history of intestinal malignancy	Steroids	Immuno‐modulators (azathioprine)	Biologics	Type of intestinal malignancy	Follow up	Death
1	M	31	42	A2B2L3L4p	No	No	No	Yes	Yes	Yes	Anal Canal Adenocarcinoma	Neoadjuvant chemoradiotherapy followed by abdominoperineal resection followed by adjuvant chemotherapy—on ustekinumab	No
2	M	30	50	A2B1L3p	No	No	No	Yes	No	Yes	Anal Canal Adenocarcinoma	Neoadjuvant chemoradiotherapy followed by abdominoperineal resection and adjuvant chemotherapy developed metastatic inguinal lymphadenopathy, underwent inguinal block dissection, later developed lung metastasis—on chemotherapy	No
3	F	29	39	A2B1L2L4p	No	No	No	Yes	Yes	Yes	Anal Canal Adenocarcinoma	Neoadjuvant chemoradiotherapy followed by abdominoperineal resection followed by adjuvant chemotherapy	No
4	F	67	71	A3B2L3	No	No	No	Yes	Yes	No	Carcinoma Left Colon	Advanced disease with hepatic metastasis	Yes
5	M	35	35	A2B2L2	No	No	No	Yes	Yes	No	Carcinoma Right Colon	Right hemicolectomy followed by adjuvant chemotherapy	Yes
6	F	32	60	A2B2L3p	No	No	No	Yes	Yes	No	Carcinoma Rectum	Total proctocolectomy with ileal pouch anal anastomosis—followed by adjuvant chemotherapy	Yes
7	M	27	31	A2B2L2	No	No	No	Yes	Yes	No	Burkitts Lymphoma Of Rectum	Anterior resection followed by chemotherapy	Yes
8	F	34	45	A2B2L4	No	No	No	Yes	Yes	No	Carcinoma Jejunum	Small bowel resection followed by chemotherapy—developed hepatic metastasis	Yes
9	M	35	59	A2B2L1	No	No	No	Yes	Yes	No	Carcinoma Ileum	Right hemicolectomy followed by chemotherapy	No
10	M	18	38	A2L3B2B3	Yes	No	No	Yes	Yes	No	Carcinoma Rectum	Subtotalcolectomy with ileorectal anastomosis followed by chemotherapy‐developed metastatic spine lesions	Yes
11	M	41	59	A2B2L3L4p	No	No	No	Yes	Yes	Yes	Carcinoma Jejunum	Small bowel resection followed by chemotherapy—developed cerebrovascular accident	Yes
12	M	49	49	A3B2L3	Yes	No	Yes	No	No	No	Carcinoma Right Colon	Subtotalcolectomy with ileorectal anastomosis—followed by adjuvant chemotherapy	No
13	F	28	43	A2B2L2	No	No	No	Yes	Yes	No	Carcinoma Rectum	Total proctocolectomy with ileal Pouch anal anastomosis—followed by adjuvant chemotherapy	No
14	M	26	48	A2B2L2	No	No	No	Yes	Yes	No	Carcinoma Rectum	Proctocolectomy with ileal pouch—anal anastomosis—on infliximab	No
15	F	74	74	A2B2L4	No	No	No	No	No	No	Carcinoma Jejunum	Small bowel resection followed by chemotherapy	Yes
16	M	35	47	A2B2B3L3L4p	No	No	No	Yes	Yes	Yes	Carcinoma Rectum	Proctocolectomy with ileal pouch—anal anastomosis‐followed by adjuvant chemotherapy—on ustekinumab	No
17	F	17	34	A2B2L3	No	No	No	Yes	Yes	No	Carcinoma Rectum	Advanced disease with hepatic metastasis	Yes
18	F	34	34	A2B2L4	No	No	No	No	No	No	Carcinoma Jejunum	Small bowel resection followed by chemotherapy	Yes

### 3.3 Cumulative Risk of Developing Bowel Malignancy

**FIGURE 2:**
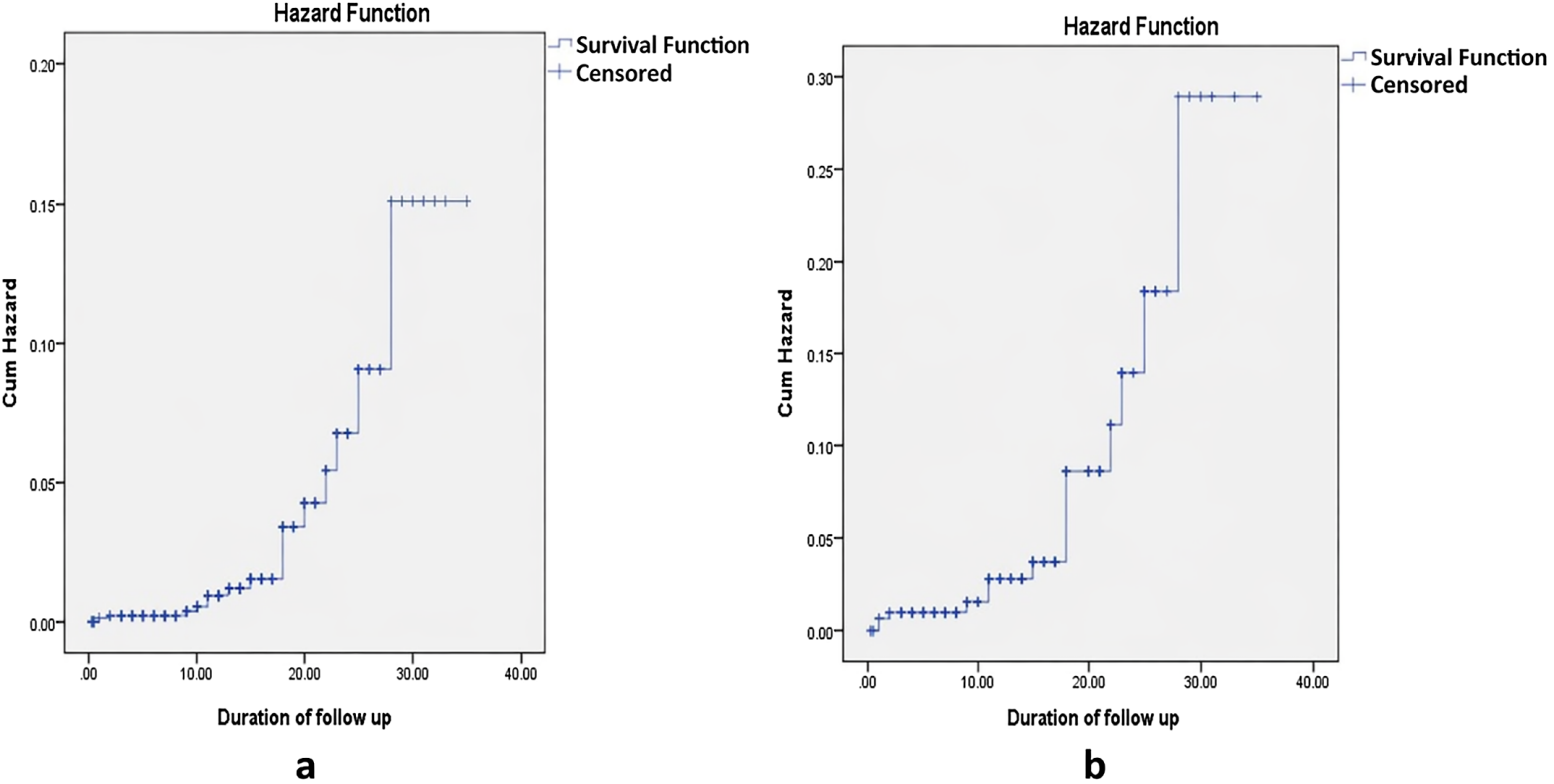
Cumulative hazard functions for incident malignancy across the follow‐up period depicting increasing incidence of intestinal malignancy with increasing duration of disease in (a) overall phenotype and (b) stricturing phenotype.

7/1416 developed malignancy during the first decade, 8/1416 during the second, and 3/1416 beyond the second decade of disease onset. The cumulative incidence of malignancy was 0.6%, 4.2%, and 14% in the first, second, and third decades of disease onset, respectively. In patients with stricturing disease, the cumulative incidence was 1.5%, 8.3%, and 25.1% across the respective decades. The cumulative hazard of developing malignancy increased with follow‐up duration, as depicted in Figure 2.

### 3.4 Risk Factors for Bowel Malignancy (Table 4a)

**TABLE 4a:** Association of various factors with malignancy in study participants.

Variable	Category	Malignancy yes	Malignancy no	*p*‐value
Age	≤ 37	1 (0.1%)	777 (99.9%)	**< 0.001**
	> 37	17 (2.7%)	621 (97.3%)	
Sex	Male	10 (1.1%)	898 (98.9%)	0.446
	Female	8 (1.6%)	500 (98.4%)	
Age of diagnosis of CD	≤ 28	5 (0.6%)	838 (99.4%)	**0.006**
	> 28	13 (2.3%)	560 (97.7%)	
Duration of CD	≤ 9.2	4 (0.5%)	839 (99.5%)	**0.002**
	> 9.2	14 (2.4%)	559 (97.6%)	
Non stricturing non penetrating	No	16 (4.2%)	367 (95.8%)	< 0.001
	Yes	2 (0.2%)	1031 (99.8%)	
Stricturing	No	2 (0.2%)	1099 (99.8%)	**< 0.001**
	Yes	16 (5.1%)	299 (94.9%)	
Penetrating	No	16 (1.2%)	1276 (98.8%)	0.667
	Yes	2 (1.6%)	122 (98.4%)	
Smoking history	No	16 (1.2%)	1373 (98.8%)	**0.044**
	Yes	2 (7.4%)	25 (92.6%)	
Family history of IBD	No	18 (1.4%)	1264 (98.6%)	0.404
	Yes	0 (0.0%)	134 (100.0%)	
Family history of bowel malignancy	No	17 (1.2%)	1381 (98.8%)	0.207
	Yes	1 (5.6%)	17 (94.4%)	
Previous ATT	No	13 (1.1%)	1224 (98.9%)	0.052
	Yes	5 (2.8%)	174 (97.2%)	

*Note:* Values shown in bold indicate *p*‐values less than 0.05, which are considered statistically significant.

Several clinical variables were evaluated for their association with intestinal malignancy in Crohn's disease (Table 4a). Increasing age was a strong determinant: patients older than 37 years had a markedly higher malignancy rate (2.7%) than those ≤ 37 years (0.1%; *p* < 0.001). Similarly, diagnosis of Crohn's disease beyond 28 years of age conferred greater risk (2.3% vs. 0.6%; *p* = 0.006). Disease duration exceeding 9.2 years was associated with a higher frequency of malignancy (2.4% vs. 0.5%; *p* = 0.002). Stricturing phenotype (5.1% vs. 0.2%; *p* < 0.001) and active smoking (7.4% vs. 1.2%; *p* = 0.044) also significantly increased risk. Together, these findings highlight that older age at onset, prolonged disease duration, stricturing phenotype, and smoking independently predispose patients with Crohn's disease to intestinal malignancy. The *strength of various covariates* with risk of developing malignancy was assessed with Cox's regression analysis (Table 4b). The following factors were seen to have an increased risk of malignancy. A HR of 6.36 (95% CI 0.8–50.6, *p* = 0.08) suggested that older age had a higher risk of malignancy, but was not statistically significant. The risk of malignancy also increased with increasing age of onset (HR 4.05, 95% CI 1.42–11.51, *p* = 0.009), stricturing phenotype (HR 14.16, 95% CI 3.19–62.85, *p* < 0.001) and active smoking (HR 7.56, 95% CI 1.70–33.57, *p* = 0.008).

**TABLE 4b:** Univariate Cox regression analysis of risk factors for malignancy in Crohn's disease.

Variable	Hazard ratio	95% confidence interval		*p*‐value
		Lower bound	Upper bound	
Age	6.363	0.8	50.596	0.08
Age at diagnosis of CD	4.048	1.424	11.509	**0.009**
Stricturing phenotype	14.162	3.191	62.848	**< 0.001**
Smoking history	7.559	1.702	33.571	**0.008**

*Note:* Values shown in bold indicate *p*‐values less than 0.05, which are considered statistically significant.

### 3.5 Subtype‐Specific Distribution of Intestinal Malignancies

#### 3.5.1 Colorectal Carcinoma

The prevalence of colorectal carcinoma in our cohort was 0.7%, with a predominance of rectal cancer (6 cases) compared to colonic cancer (3 cases). The cumulative probabilities of developing colorectal carcinoma were 0.62% at 10 years, 1.58% at 20 years, and 6.20% beyond 20 years after Crohn's disease diagnosis. When patients with isolated small bowel involvement were excluded, the risk increased marginally to 0.70%, 1.75%, and 6.97% at the same time points. Only two of six rectal cancer patients (33.3%) had a history of chronic perianal fistula, and this association was not statistically significant (*p* = 0.41).

#### 3.5.2 Small Bowel Carcinoma

Small bowel carcinoma occurred in 0.35% of the cohort, corresponding to a cumulative incidence of 35.3 per 10 000 patients. The jejunum was the most frequent site of involvement. Malignancy risk was significantly higher in patients with small bowel–limited disease (*p* < 0.001) and in those with a stricturing phenotype. All affected patients succumbed within 1 year of diagnosis.

#### 3.5.3 Anal Canal Carcinoma

Three patients (0.21%) developed anal canal carcinoma, all histologically confirmed as adenocarcinoma. Each of these patients had chronic perianal fistulizing disease with anal canal stricture (3/22, 13.6%). The mean interval from Crohn's disease diagnosis to cancer development was 13.7 ± 5.5 years.

## 4 Discussion

Eighteen (1.27%) patients developed intestinal malignancy in a mean disease duration of 15.9 ± 10.3 years. The incidence density of intestinal malignancy was1.379/1000 person‐years which would mean an annual incidence of 0.13%. In a recent study by Sharma et al. [11] from northern India including 952 individuals with CD, the incidence of intestinal malignancy was 0.42% and the incidence density of intestinal malignancy was 0.86/1000 person‐years. The cumulative risk of developing malignancy was 0.6%, 4.2% and 14% in the first, second and third decade of disease onset respectively while in our study it rose steadily over time, reaching about 14% overall and 25% in stricturing disease by the third decade—paralleling the pattern seen in Sharma et al. [11]. The distribution of malignancy sites revealed that colorectal malignancy was the most frequent, accounting for 10 of 18 cases (55.5%), followed by jejunal carcinoma in 4 cases (22.5%), anal canal carcinoma in 3 cases (16.5%), and ileal carcinoma in a single patient (5.6%). The prevalence of CRC in our study was 0.7%, similar to the observation by Uchino et al. [7]. An increased risk of rectal cancer (six patients) compared to colon cancer (three patients) seen in our study align with other Asian studies [12, 13] while the reverse was true in Western studies [14, 15, 16]. The cumulative probability of developing colorectal carcinoma in our cohort was 0.62% at 10 years, 1.58% at 20 years, and 6.20% beyond 20 years after Crohn's disease diagnosis, compared with 0.3%, 3.8%, and 3.8% at 10, 20, and 30 years, respectively, in the Korean study by Lee et al. [17]. When patients with isolated small bowel disease were excluded, these probabilities increased to 0.70%, 1.75%, and 6.97%, versus 3.0%, 4.7%, and 4.7% in the corresponding Korean cohort [17]. Among the 6 CD patients with rectal adenocarcinoma, only 2 (33.33%) had a history of chronic perianal fistula unlike the Korean study [17] where more than 70% of rectal cancer patients had chronic perianal fistula. The cumulative probability of rectal cancer was higher in CD patients with a perianal fistula than without (*p* = 0.02) in the same study [17] while it was not statistically significant in ours (*p* = 0.41).

The cumulative incidence of SCA in CD was 35.3 per 10 000 in our study while it was 12.3 per 10 000 (95% CI 9.2–16.3 per 10 000) in a study from Scandinavia by Yu et al. [18]. The prevalence rate of SCA in our cohort was 0.35%, lower than 1.6% reported in a study from the United States by Shaukat et al. [19] and 1.15% in a meta‐analysis by Chin et al. [20]. We saw an increased risk of developing SCA in patients with disease limited to the small bowel (*p* < 0.001) as reported by von Roon et al. [21]. An increased risk for SCA was seen in stricturing small bowel disease as observed by Axelrad et al. [22]. All patients with SCA expired within 1 year of diagnosis in our cohort which is similar to the observation by Axelrad et al. [22] where they found that the HR for SBC death was higher among patients with CD during the first year of follow up.

Three patients developed carcinoma in anal canal in our cohort with a point prevalence of 0.21%. All the patients with anal canal carcinoma in our cohort had anal canal stricture with chronic perianal fistulizing disease, that is, 3 out of 22 patients (13.6%), while in a series by Brochard et al. [23], two patients (2%) developed anal adenocarcinoma in a series of 102 patients with anal canal stricture on follow‐up.

The *risk factors* for intestinal malignancy in CD have been looked into in a number of studies. They included duration of disease, disease phenotype, coexisting PSC, family history of intestinal malignancy, smoking status, prior surgeries, and age of onset. In our cohort, the risk of intestinal malignancy rose with longer disease duration, particularly among patients with a stricturing phenotype, as demonstrated by Kaplan–Meier analysis. The cumulative probability of malignancy in stricturing disease reached 25% by the third decade after diagnosis, markedly higher than that observed within the first decade. A later age of Crohn's disease diagnosis was also an independent predictor, with patients diagnosed after 28 years showing a substantially greater risk of intestinal malignancy. In a study from Sweden by Ekbom et al. studying the risk of CRC in CD [24], a higher relative risk was observed in those in whom CD was diagnosed in less than 30 years than those diagnosed at an older age, and duration of disease follow‐up did not affect the relative risk of CRC in CD, while Axelrad et al. [22] in their study identified that the risk of SBC increased with age of diagnosis of disease. Stricturing phenotype was associated with an increased risk of small bowel and colorectal malignancy. This observation is consistent with findings by Axelrad et al. [22], who also reported a higher incidence of small bowel carcinoma in patients with stricturing disease. Active smoking was associated with increased risk of intestinal malignancy in our cohort. One hundred seventy‐nine patients in our cohort had taken empirical anti–tuberculous therapy (ATT) at the onset to differentiate CD from intestinal tuberculosis. Of the above, 58, that is, 32.4% developed stricturing disease on follow‐up, which was also seen in the study by Gupta A et al. [25] where they found that empirical ATT predisposed to intestinal strictures. Prior ATT was not associated with increased risk of intestinal malignancy in our study. No patient had primary sclerosing cholangitis (PSC) in our cohort. Both family history of IBD and family history of bowel malignancy were not associated with increased risk of intestinal malignancy in our study. No medications were associated with increased risk of intestinal malignancy in our cohort.

This is one of the few studies from the Indian subcontinent which looked into the prevalence and risk factors of intestinal malignancy in CD. The SIR of intestinal malignancy in our CD cohort was 95.36 (95% CI: 52.98–143.04) indicating that the observed risk of intestinal malignancy is approximately *95 times* higher than expected based on general population rates. In a recent population‐based cohort study from China, there was no significant cancer risk between CD patients and the general population [26], while a study from Denmark [27] showed increased risk of colonic and small bowel malignancy in CD compared to the general population—a finding corroborated by our study.

There are a few limitations in our study. The main limitation is the inability to accurately pinpoint the date of CD onset. In our study, two cases of jejunal carcinoma and two cases of CRC were diagnosed at the onset of CD diagnosis. If we accept that malignant transformation in CD usually takes place in a few years, then the delay in diagnosing could be attributed to the minimal symptoms or patients being totally asymptomatic, especially in stricturing or small bowel predominant disease, and the development of malignancy creates a critical stricture whereby the patient presents with intestinal obstruction or perforation. Secondly, this being a hospital‐based study conducted at a tertiary referral center, with a high proportion of stricturing and perianal phenotypes—both strongly linked to intestinal cancers—which legitimately inflates risk versus population cohorts. Also, intensive endoscopic and radiological surveillance in tertiary centers can lead to greater case ascertainment of malignancies that may remain undetected in population registries, thereby increasing the observed‐to‐expected ratio. The center also functions as a high‐volume tertiary surgical IBD unit, receiving disproportionate referrals for stricturing, penetrating, fistulizing, perianal and post‐operative Crohn's disease. This enriched case mix, combined with long disease duration, naturally concentrates phenotypes known to carry the highest cancer risk, thereby elevating the observed SIR relative to population‐based cohorts. Thirdly, the SIR calculation is based on *crude national incidence rates* for the general population, which includes individuals without Crohn's disease and with shorter life expectancy or differing age distributions. This may underestimate the “expected” number of cancers, thereby amplifying the SIR. Additionally, the small number of observed cases magnifies the relative variance, resulting in a wide CI and an apparently inflated point estimate. Fourthly, although data was recorded prospectively, the analysis was retrospective in nature which led to loss to follow up in some participants; these participants were censored at their last documented visit, with disease duration calculated accordingly. Lastly, we didn't have a non‐CD control group, the absence of which was mitigated by referencing general population cancer rates from the study by Satishkumar et al. [10]. This approach mitigates the absence of an internal control arm but may have contributed in part to the high SIR observed, as population‐based rates likely underestimate the true background risk in comparable tertiary referral populations.

In summary, there exists a definite risk for intestinal malignancy in CD in the Indian population. Our findings reaffirm that malignant transformation predominantly occurred in strictured segments, which underscore the need for focused surveillance of stricturing Crohn's disease, particularly in long‐standing cases. Although there are guidelines for cancer surveillance in colonic IBD, the small bowel and perianal region are not easily amenable to preventive strategies. Future research and guidelines should help to formulate screening strategies in these cases.

## Funding

The authors have nothing to report.

## Conflicts of Interest

The authors declare no conflicts of interest.

## Data Availability

The data that support the findings of this study are available from the corresponding author upon reasonable request.
